# Internal mammary lymph node metastasis in breast cancer patients based on anatomical imaging and functional imaging

**DOI:** 10.1007/s12282-022-01377-7

**Published:** 2022-06-24

**Authors:** Wei Wang, Pengfei Qiu, Jianbin Li

**Affiliations:** 1 Department of Radiation Oncology, Shandong Cancer Hospital and Institute, Shandong First Medical University and Shandong Academy of Medical Sciences, 440 Jiyan Road, Jinan, 250117 China; 2 Breast Cancer Center, Shandong Cancer Hospital and Institute, Shandong First Medical University and Shandong Academy of Medical Sciences, Jinan, 250117 Shandong Province China

**Keywords:** Internal mammary lymph node, Metastasis/recurrence, Preoperative anatomical imaging, Preoperative functional imaging, Postoperative pathological findings

## Abstract

Internal mammary lymph node (IMLN) metastasis forms part of the clinical node classification for primary breast cancer, which influences the treatment strategy. However, because of the IMLNs’ complicated anatomical structures and relationships with adjacent structures, IMLN biopsy or resection is associated with a limited improvement in prognosis and a high complication rate. The positivity rate also varies broadly according to imaging modality, and there is a low rate of agreement between the imaging and pathological diagnoses, which creates imprecision in the preoperative staging. The IMLN positivity rate also varies remarkably, and there are no clear, accurate, and non-invasive modalities for diagnosing the pre-mastectomy IMLN status. Nevertheless, medical imaging modalities continue to evolve, with functional imaging and image-guided thoracoscopic biopsy of sentinel IMLNs being well established. Thus, personalized decision-making and treatment selection should be based on the modality-specific differences in the diagnosis of IMLN metastasis/recurrence and the patient’s specific risk factors.

## Background

Early clinical trials have shown that the internal mammary lymph node (IMLN) status is an important prognostic factor for breast cancer, and knowledge of the IMLN status is essential to guide the treatment strategy [1–4]. However, recently clinical trials have indicated that IMLN metastasis does not independently predict overall survival (OS) and progression-free survival (PFS) for patients who receive personalized treatment (e.g., chemotherapy, endocrine treatment, targeted treatment, and radiotherapy) [5–9]. Thus, accurate staging plays a significant role in guiding effective, multidisciplinary, and personalized treatment for newly diagnosed breast cancer patients. The American Joint Committee on Cancer (AJCC) staging guidelines include detailed clinical stages for patients with breast cancer [10], although the IMLNs’ location is relatively deep within the chest wall and in the parasternal intercostal spaces, which precludes palpation during a clinical examination. Moreover, the complex anatomical structures in this region create differences in the rates of preoperative imaging-based IMLN diagnosis and histopathologically confirmed IMLN metastasis. For patients with breast cancer (stage I, II, or III), observed IMLN recurrence rate was < 1.5% after primary breast cancer treatment, even when the internal mammary chain (IMC) not excised or irradiated [11–13]. Patients most likely benefit from systemic therapies and incidental regional node irradiation [14–18]. But according to extended radical mastectomy data, 9.2% of patients with no positive axillary nodes (ALN) present IMLN metastasis [19]. The current indications for IMLN irradiation might result in over-/under-treatment according to the National Comprehensive Cancer Network (NCCN) clinical practice guidelines (Version1. 2016) and the Royal College of Radiologists (RCR) consensus statement [20, 21]. These issues have generated controversy regarding the clinical diagnosis of IMLN metastasis in breast cancer patients, which is required to guide the use of local treatment such as radiation therapy and surgery. Therefore, we have reviewed the detection of IMLN metastases/recurrence based on various imaging modalities, as well as its risk factors and prognostic characteristics.

## IMLN metastasis in newly diagnosed patients

### Preoperative anatomical imaging

**Fig. 1:**
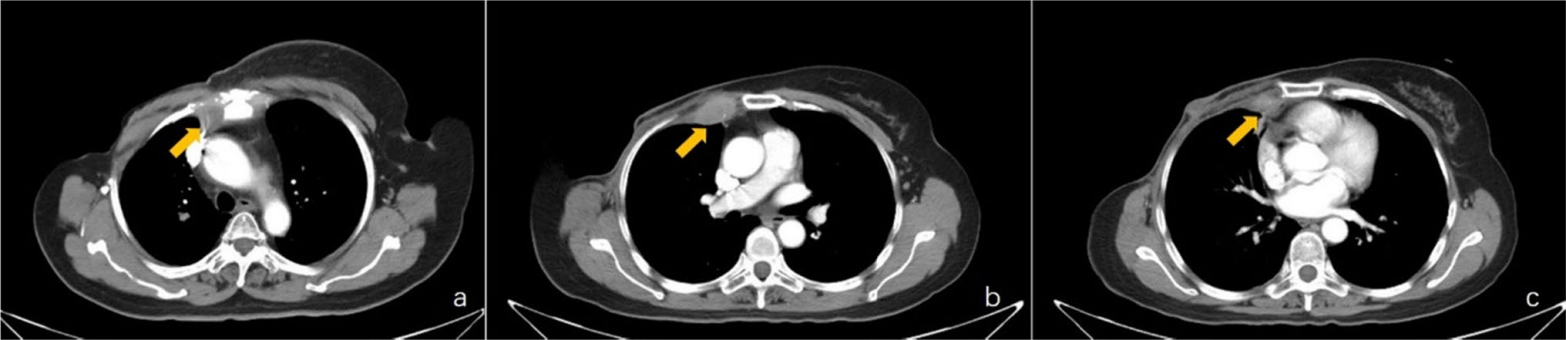
Metastatic internal mammary lymph nodes (IMLNs, yellow arrows) detected during computed tomography. A metastatic IMLN in the first intercostal space (**a**), a metastatic IMLN in the second intercostal space (**b**), and a metastatic IMLN in the third intercostal space (**c**)

**Fig. 2:**
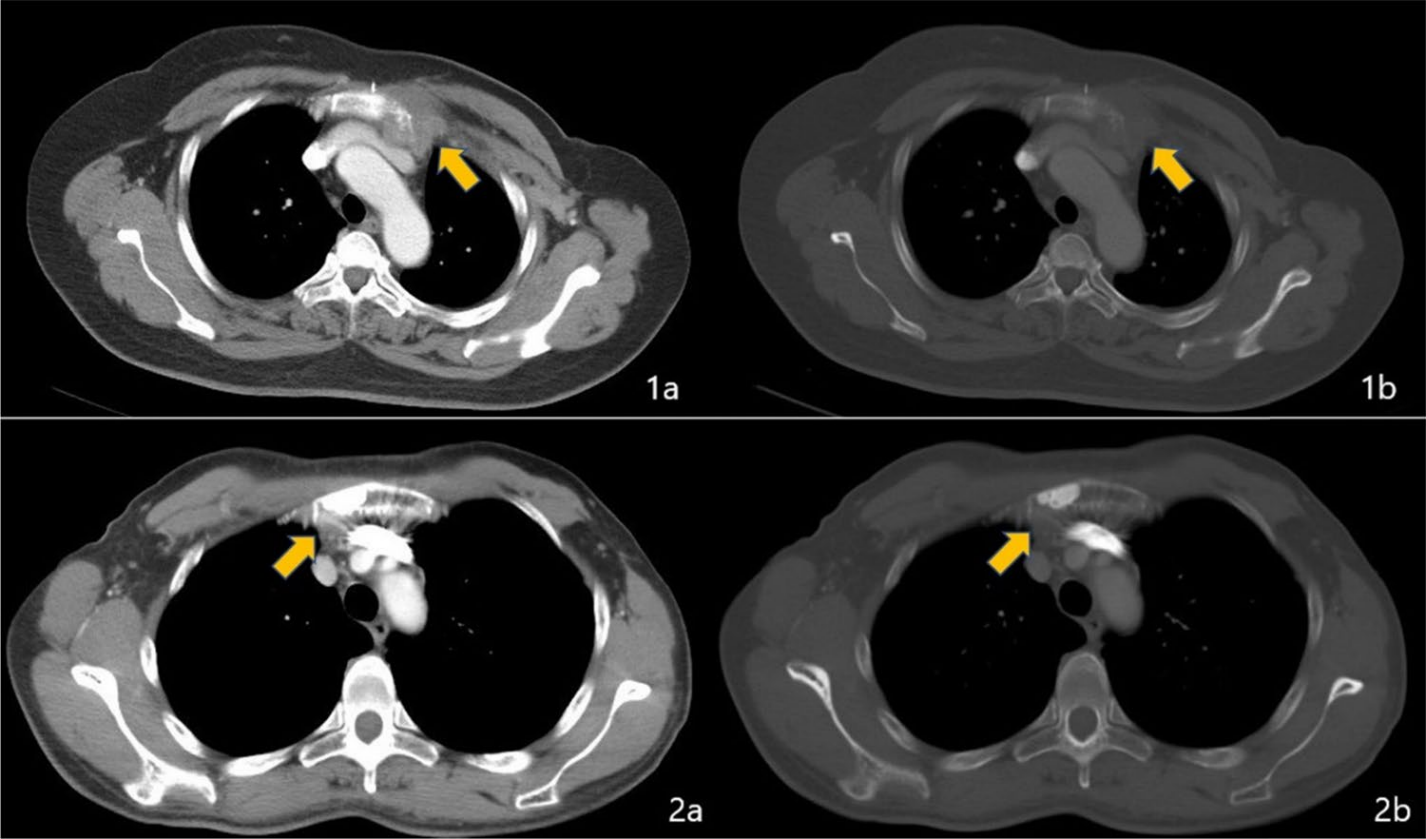
Computed tomography reveals internal mammary lymph node metastasis (yellow arrow) with sternal erosion, sternal metastasis (yellow arrow), and a local soft tissue lump. Views are shown in the mediastinal window (**a**) and bone window (**b**)

Preoperative anatomical imaging is a non-invasive technique that can be used to identify positive IMLNs, which can be performed using ultrasonography (US), computed tomography (CT), and magnetic resonance image (MRI) (Table 1). Swollen lymph nodes (LNs) are a characteristic sign of IMLN metastasis on CT images (Fig. 1), with contrast-enhanced CT identifying positive IMLNs in nearly 42% of patients with breast cancer, and > 50% of the identifiable IMLNs were > 5 mm in size [22, 23]. However, partial IMLN metastasis with sternal erosion or osteolytic sternal metastasis with a local soft tissue lump have some resemblance to swollen IMLNs with sternal erosion [24], which can make it difficult to diagnose IMLN metastasis based on CT images (Fig. 2). The rate for pathological confirmation of IMLN metastasis rate was 57% among all imaging-positive patients who received neo-adjuvant chemotherapy (NAC), with rates of 74% among US-positive patients and 70% among MRI-positive patients [6], which provided superior sensitivity and specificity, relative to CT.

**Table 1:** Summary of IMLN metastasis accuracy of the various imaging techniques

	No. of patients	No. of IMLN	Sensitivity	Specificity	Accuracy
US [6]	114	70	74%	53%	78.57%
MRI[29]	16	43	93.30%	89.30%	90.7%
PET/CT [41]	249	31	87.10%	–	–
PET/MRI [44]	80	9	88%	100%	88.89%
SPECT/CT combined lymphoscintigraphy (IM-SLN) [86]	211	148	96.70%	97.70%	98.70%

Jeong et al. [25] explored the shape and depth of metastatic IMLNs using US, which revealed that 85% of the metastatic IMLNs had an oval shape and the other 15% had an irregular shape, with positive IMLNs typically being found in the posterior aspect of the intercostal spaces. Among newly diagnosed breast cancer patients, the prevalence of positive IMLNs based on US was 10%, and US-guided needle biopsy confirmed malignancy in 90% of these cases, with 1.3% of the patients having isolated IMLN metastasis. Younger patients had an increased risk of IMLN metastasis. Internal mammary (IM) US has been used to change the N classification for 8% of patients and to change the overall clinical stage for 6.4% of patients, which ultimately necessitated a change in the treatment strategy for some patients [26]. However, approximately 40% of those patients underwent NAC before surgery, which may have prevented micrometastatic IMLNs from being identified at the LN biopsy, and subsequently reduced the positivity rate. Furthermore, the IMLNs cannot be easily identified using US in patients with a high body mass index (BMI) or thick muscle and intermuscular fat. Moreover, perforating branches of the internal mammary veins (IMVs) can easily be confused with IMLNs during US, although color Doppler US can distinguish an IMLN from a perforating branch of the IMVs based on the blood flow ultrasonic signal [27].

**Fig. 3:**
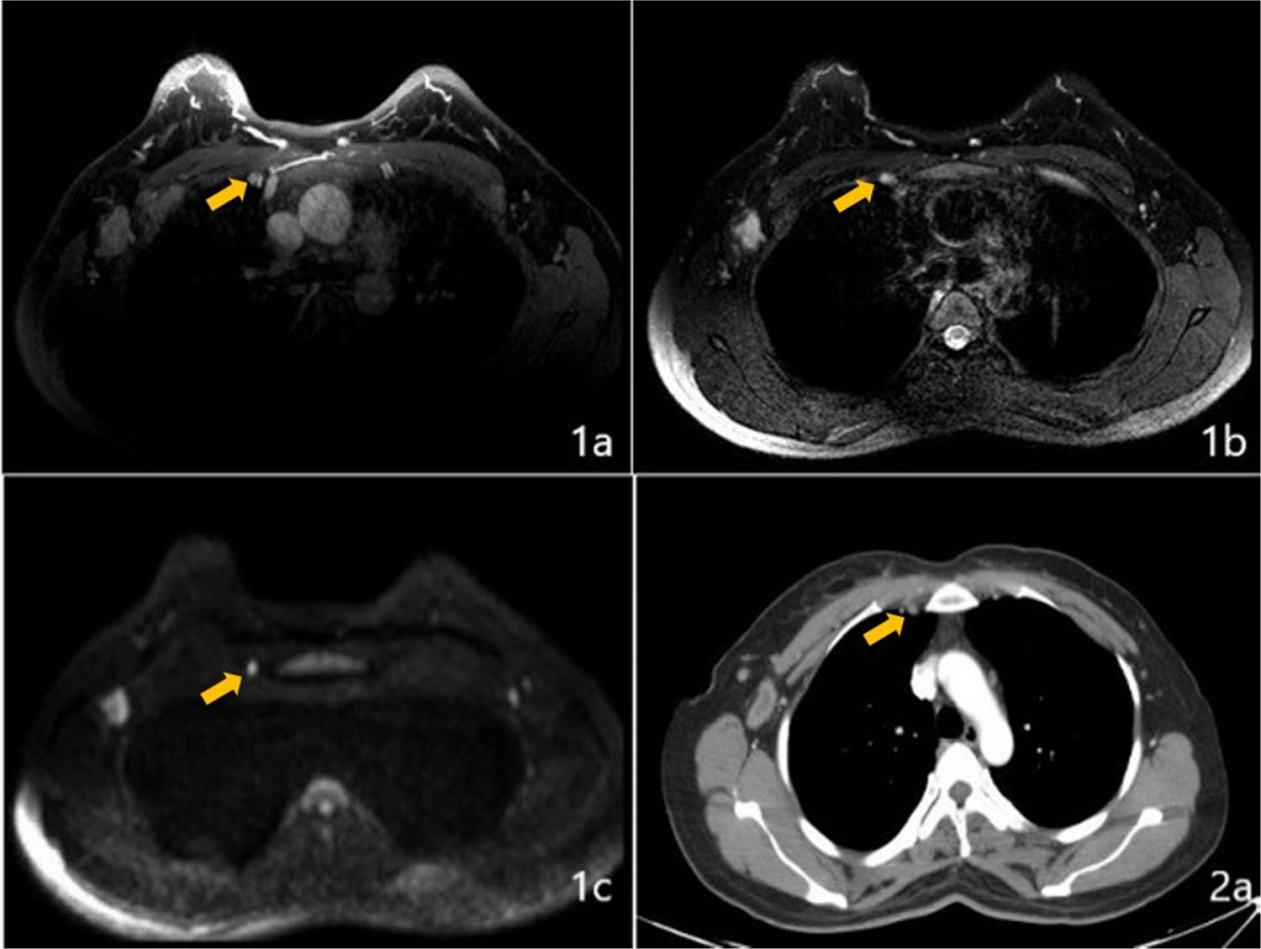
A metastatic internal mammary lymph node (yellow arrow) detected during using dynamic contrast-enhanced T1-weighted magnetic resonance imaging (**1a**), T2-weighted imaging (**1b**), diffusion-weighted imaging (**1c**), and computed tomography (**2a**)

Preoperative anatomical imaging can support a diagnosis based on the changing shape and size of LNs, as well as other imaging features, although both physiologic and metastatic IMLNs enhance during dynamic contrast-enhanced breast MRI. Sachdev et al. [28] reported that only 0.3% of breast cancer patients had positive IMLNs that were identified at the pre-treatment MRI, with 96% of positive nodes being located in the first two intercostal spaces and the remaining 4% located in the third intercostal space. For high-risk patients and using a size threshold of 4.5–5 mm, MRI provides good sensitivity (93.3%) and specificity (89.3%) for diagnosing metastatic IMLN [29, 30]. However, clear and universally recognized standards for identifying IMLN metastasis are lacking. Patel et al. [31] analyzed the before and after MRI findings of suspicious IMLNs in breast cancer patients who were receiving neo-adjuvant therapy, and suggested that metastatic IMLNs should be suspected when the diagnostic MRI reveals ≥ 3 ipsilateral IMLNs that are ≥ 6 mm. However, this approach will have an increasing false-negative rate at smaller tumor sizes. The IMV and metastatic IMLN both exhibit high signal intensities during diffusion-weighted MRI, which suggests that using multiple MRI parameters and sequences may help improve the diagnosis rate (Fig. 3).

### Preoperative functional imaging

**Fig. 4:**
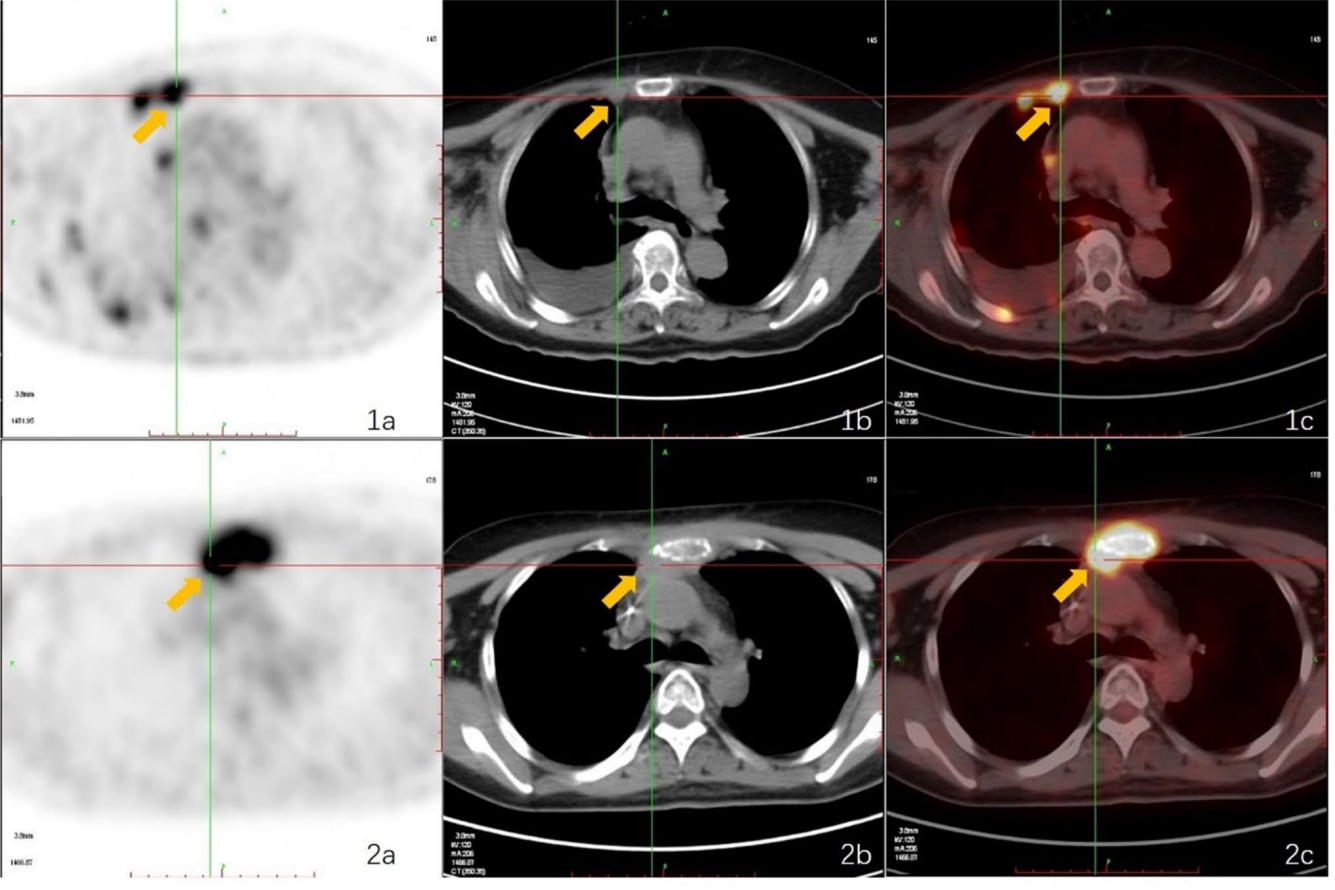
Internal mammary lymph node metastasis (yellow arrow) with sternal erosion detected based on FDG uptake (**1a**), computed tomography (**1b**), and a fused FDG PET/CT image (**1c**), as well as sternal metastasis (yellow arrow) with a local soft tissue lump detected based on FDG uptake (**2a**), computed tomography (**2b**) and a fused FDG PET/CT image (**2c**)

Relative to standard anatomical imaging, positron emission tomography (PET) combined with CT or MRI (PET/CT or PET/MRI) improves the ability to detect positive IMLNs in breast cancer patients [32–39]. Furthermore, use of PET provides a positive predictive value of > 80% [40, 41] (Fig. 4), even for patients who received NAC, and the pathological confirmation rate was up to 55% in PET-positive patients [6]. However, some trials have indicated that ≤ 10% of breast cancer patients have positive IMLNs based on PET-CT [40, 42]. In addition, subgroup analysis revealed that disease stage significantly influenced the likelihood of IMLN metastasis, with positive IMLNs relative proportion within all LN region metastases (level I–III, the supraclavicular region, and the internal mammary region) were 3.7% in primary M0 patients and 9.5% in M1 patients [42]. Inflammation, infection, and reactive hyperplasia may create a false-positive result for FDG uptake at the IMLNs during PET/CT.

Based on better soft tissue contrast and motion correction possibilities [43], compared to PET/CT, statistics display a definite trend toward lower specificity and higher sensitivity of PET/MRI in the lesion-by-lesion analysis [44, 45]. Moreover, PET/CT or PET/MRI are currently available only at select hospitals, and these modalities are more expensive than more traditional modalities.

### Histopathologically confirmed IMLN metastasis

Anatomical imaging (US, CT, and MRI) and functional imaging (PET/CT, PET/MRI, and lymphoscintigraphy) reportedly provide high sensitivity and accuracy for diagnosis of IMLN metastasis [6, 29, 30, 40–42]. However, a pathological diagnosis (are intercostal space IMN biopsy or endoscopic lymphatic chain resection) is still important for determining IMLN metastasis, as the pathological LN stage may change in a subset of patients, who may then require a revised treatment strategy [9, 46]. Although the pathological determination of IMLN status remains important, the performance of intercostal space IMN biopsy and endoscopic lymphatic chain resection remains controversial because these techniques are not associated with improvements in survival or in intraoperative/postoperative complications [13, 47, 48]. Thus, IMLN dissection is not routinely recommended.

When using a free flap for immediate autologous reconstruction after mastectomy, the internal mammary artery (IMA) and IMV are the preferred recipient vessels because of their large caliber and high flow rate, which provides a reasonable opportunity to study IMLN metastasis. Recent studies have identified that 3.6–6% of breast cancer patients have positive IMLNs based on biopsy performed during immediate reconstruction after mastectomy [48–50]. However, among 2057 patients who underwent free-flap breast reconstruction, Ochoa et al. [51] found that only 28 patients (1.3%) had positive IMLNs, and the preoperative breast MRI provided 11% sensitivity for detecting the IMLN. Ten of the 28 patients with IMLN metastasis (36%) had nodal metastases that were isolated to the IMLNs, and isolated IMLN involvement was associated with a lower T classification.

While difficult relative to axillary sentinel LN biopsy, the success rate for IMLN biopsy currently averages 90% for experienced teams [52–55]. Thoracoscopic IMLN dissection in breast cancer has also been reported with the same positive IMLN rate and less morbidity than the conventional intercostal space incision [9, 56]. In addition, image-guided (especially preoperative lymphoscintigraphy single-photon emission computed tomography (SPECT) with CT (SPECT/CT)-guided) thoracoscopic biopsy of the sentinel IMLNs can further improve the detection rate [5, 13, 57–59]. Piato et al. [60] found that 15% of patients had a change in the pathological LN staging when lymphoscintigraphy was performed after the intra-tumor technetium-99 m injection. Results from a prospective study also showed that 21% of early-stage breast cancer patients had IMC drainage identified based on lymphoscintigraphy, although only 13% of these patients had pathologically confirmed IMLN metastasis after IM-SLN biopsy. Moreover, there was no difference in recurrence-free survival between the patients with a positive IMLN and the patients without an IMC-sensitive node biopsy [13]. Using the same techniques, a multicenter cohort study revealed that 20.5% of ipsilateral IMLNs were visualized during preoperative lymphoscintigraphy, while only 3.53% of patients were found to have IMLN metastasis [5]. The presence of IMLN metastasis was also associated with ALN metastasis [5, 61], and the same relationship was observed among patients who had received NAC [59].

## Minimally invasive diagnosis of IM-SLNB

**Fig. 5:**
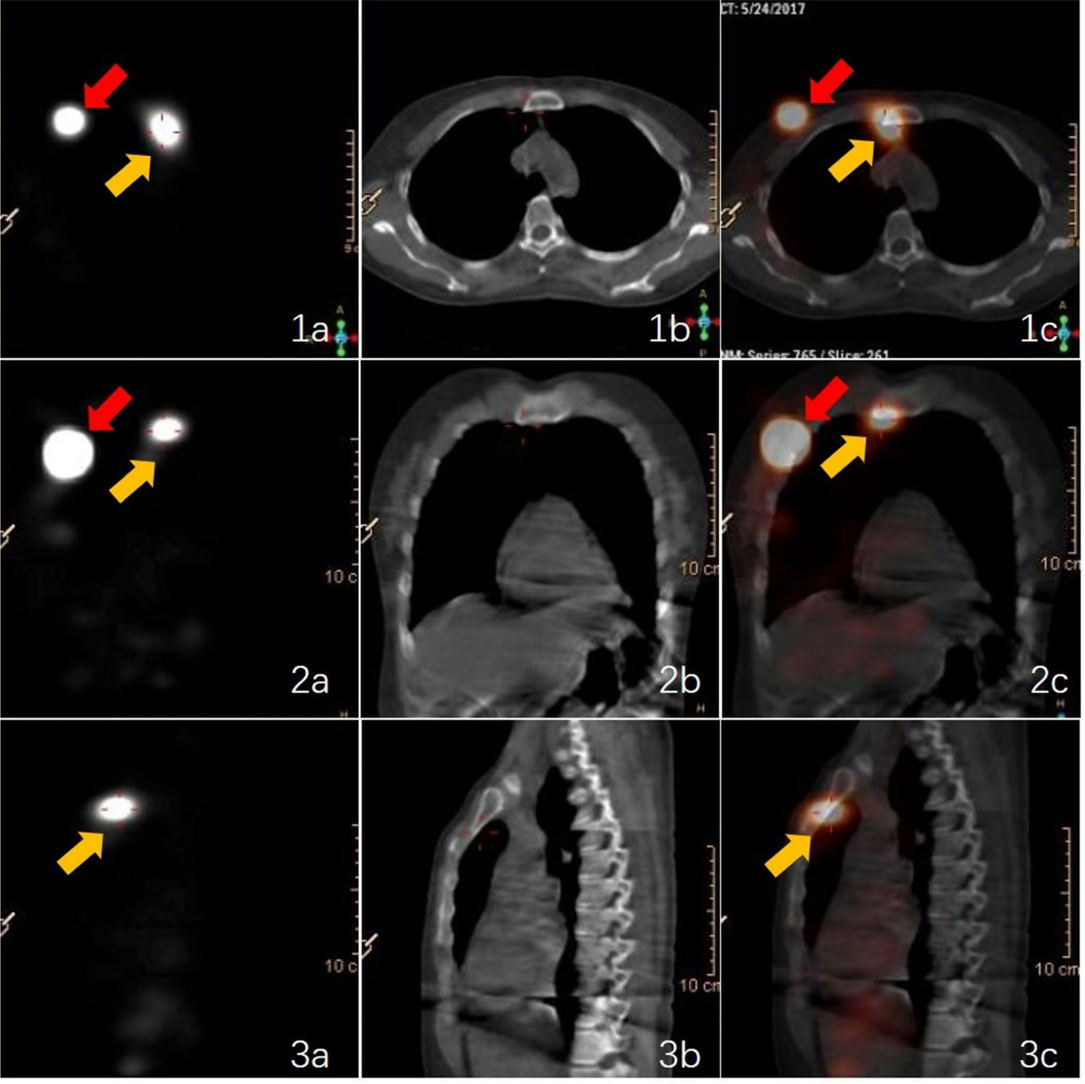
A positive internal mammary lymph node (yellow arrow) detected using single-photon emission computed tomography (**a**), computed tomography (**b**), and SPECT/CT (**c**) viewed in the axial plane (1), coronal plane (2), and sagittal plane (3). The red arrow indicates the injection point

Compared to extended surgical resection, IM sentinel lymph node biopsy (IM-SLNB) provides a less invasive method of assessing the IMLN. However, low visualization rate of IM-SLN has been a restriction of IM-SLNB. Routine SPECT can help identify lymphatic drainage and the form, size, amount, and distribution of the sentinel lymph nodes (SLNs) based on tracer drug uptake. However, precise identification of the IMLNs and clarification of their relationships with the surrounding structures are also important. Hybrid SPECT/CT can provide complementary functional and anatomical information (Fig. 5), as well as mapping of lymphatic drainage via three-dimensional (3D) reconstruction. Relative to lymphoscintigraphy alone, SPECT/CT provides advantages in terms of accurate anatomical localization, identification of false-positive results, fewer false-negative results, and better guidance regarding the surgical approach. Using lymphoscintigraphy and SPECT/CT after an intra-lesion injection of 99mTc-nanocolloid, the sentinel IMLN was found to contain metastasis in only 24% of patients from a pilot study [60] and in 34% of patients from a Dutch multicenter study [55]. The Breast Cancer Center of Shandong Cancer Hospital optimized the use of 99mTc-labeled sulfur colloid (99mTc-SC) in sentinel IMLN mapping and detection, evaluated the SPECT-CT image acquisition time, and modified the injection technique (peri-areolar intraparenchymal, high volume, and ultrasound guidance), which increased the IM-SLN visualization rate to 71.9% for breast cancer patients who were receiving initial surgery [52, 57, 58, 62, 63]. Furthermore, among patients who underwent NAC, the IM-SLN visualization rate was 33.1% [52, 62]. An acoustic probe commonly captures diffuse activity along the rib cage and care is needed when radioactive IMLNs are present in multiple intercostal spaces, as the sentinel and non-sentinel IMLNs can easily be confused [60]. The sentinel IMLNs near the radiotracer injection site are also easily ignored, which can lead to an increase in the false-negative rate. Finally, the use of NAC can influence the basal metabolic rate, radiotracer injection concentration, and tumor staging, which can lead to inter-patient differences in the radiotracer’s uptake and metabolism [64]. These differences would presumably translate into variability in the detection rate.

## IMLN recurrence after treatment

IMLN recurrence after systematic breast cancer treatment is poorly characterized. The 5-year OS in patients with IM and/or supraclavicular (IM–SC) LN recurrence without distant metastasis (DM) was 51% compared with 27% in patients with DM recurrence. The rate of IM–SC LN recurrence without DM was 0.3%, and with DM was 6.5% at the first recurrence [65]. Median OS for patients with IMN recurrences as a first event was 2.5 years, with 5 year OS 28% [66]. Sachdev et al. [28] reported that, after a median follow-up of 38 months for 7070 breast cancer patients who did not undergo IMC resection, loco-regional control could be achieved using IMC irradiation combined with modern systemic therapy. Based on FDG-PET/CT-positive loco-regional LN metastases (defined as the ALNs, supraclavicular LNs, and IMLNs), the rates of LNs in the IMC region were 3.7% for patients with primary M0 cancer and 9.5% for patients with primary M1 cancer, while the rates increased to 9.4% for patients with recurrent M0 cancer and 11.8% for recurrent M1 cancer [42]. Regardless of the imaging modality, follow-up results have indicated that the overall rate of clinically detectable IMLN recurrences remains < 1.5% after primary systemic breast cancer treatment, even when the regional IMLNs were not irradiated [12, 67–71] (Table 2). In addition, the IMLN recurrence rate was reduced to 0.2% for patient who had undergone IMLN radiotherapy (IMLN RT) [12]. Similar to in primary breast cancer patients, the recurrent IMLNs were still concentrated in the second intercostal space (67.7%) and the third intercostal space (19.5%) [68]. Loganadane et al. [71] evaluated the loco-regional failure patterns in 796 women with breast cancer who underwent irradiation of the chest wall ± the supraclavicular region (level IV, 88.3%), ± the infraclavicular region (levels II–III, 77.9%), ± the IMC region (85.6%), ± the ALN region (level I, 14.9%). During a median follow-up of 64 months (range: 6–102 months), only one patient developed IMLN recurrence, which occurred within the irradiated volumes. LN metastasis was observed in 17.5 and 31.5% of cases when irradiation tagret delineation was based on the RTOG and ESTRO guidelines, respectively. As geographic misses outside the ESTRO-clinical target volume (CTV) while within the RTOG-CTVs occurred in 14% of the cases. This may be because the ESTRO-recommended volumes were designed for early-stage breast cancer, while the RTOG guidelines are probably more suitable for advanced tumors [71–73]. Therefore, part of the loco-regional failure after radiotherapy may be related to poor tumor targeting. So, tailored target delineation guideline should be selected.

**Table 2:** The overall rate of IMLN recurrences after primary systemic treatment

Author	Year	No. of patients	IMLN recurrence (%)
Cranenbroek et al. [67]	2005	5912	0.1
Chen et al. [68]	2010	8867	1.5
Ohsumi et al. [69]	2011	1907	0.26
Oh et al. [70]	2014	1906	1.47
Poortmans et al. [12]	2015	4004 (all)	0.5
		2002 (nodal-irradiation group)	0.2
		2002 (thoracic wall irradiation only group)	0.8
Loganadane et al. [71]	2017	796	0.13

Follow-up results have shown that benign or malignant IMLNs can be detected using MRI in 37.6% of breast cancer patients who underwent silicone implant-based oncoplastic surgery, with median short-axis and long-axis measurements of 0.40 and 0.70 cm. However, the surgical and percutaneous biopsy results revealed that only 0.48% of the IMLNs (1/207) were malignant, which corresponded to a positive predictive value of 0.5% for predicting malignancy based on MRI-detected enlarged IMLNs [74]. The high rate of false-positive IMLN metastasis, which were incorrectly identified during preoperative imaging and reviewed after breast cancer surgery, may be related to several factors. First, inflammation or LN reactive hyperplasia may be a confounding factor. Second, patients who receive silicone implants have increasing risks over time of silicone migration and possibly silicone granulomatous lymphadenitis [75, 76]. Third, some studies have shown that lymphatic drainage patterns might be altered by lymph vessel shrinkage, fibrosis, and obstruction caused by cellular material or tumor emboli, especially for patients who have received chemotherapy [77, 78]. Fourth, various tissues can mimic IMLNs during parasternal imaging, such as mature adipose tissue, costal cartilage, part of a vessel, skeletal muscle, or fibroblasts [25]. Finally, granulomatosis or acute infective pleurisy are also potential confounding factors [79]. Therefore, for patients with large IMLNs or functional imaging-positive IMLNs after surgery, it may be more appropriate to use a short-interval follow-up with enhanced CT, MRI, US, or biopsy, rather than an immediate full oncologic work-up, depending on the size and location of the IMLNs.

## Predictive and prognostic factors of IMLN metastasis

Many studies have investigated the relationships between IMLN metastasis and various clinical, pathological, and immunohistochemical parameters. Among newly diagnosed breast cancer patients, IMLN metastasis is associated with age, tumor location/depth, and the tumor’s expression of estrogen receptor (ER), progesterone receptor (PR), and human epidermal growth factor receptor-2 (HER-2) [2, 9, 40, 50, 80–85]. For example, Heuts et al. [2] reported that the proportions of IMLN metastasis were 32% for < 50-year-old patients, 15% for 51- to 70-year-old patients, and 11% for > 70-year-old patients in their cohort. Huang et al. [81] also reported that the proportions of IMLN metastasis were 21.8% for < 35-year-old patients, 15.7% for 35- to 50-year-old patients, and 12.9% for > 50-year-old patients in their cohort. Thus, younger patients appear to have a higher risk of IMLN metastasis. The tumor was located in the central or medial breast in 60% of patients with proven positive IMLNs [2], while tumors located in the upper outer quadrant had a smaller likelihood of having positive IMLNs (approximately 10%) [83]. Patients with negative ER expression were 2.8 times more likely to have positive IMLNs, relative to patients with positive ER expression, based on findings from FDG-PET/CT [40], and other multivariate analyses have revealed that IMLN metastasis was independently associated with positive HER-2 expression [9, 50, 85]. Furthermore, the risk of IMLN metastasis increased at higher numbers of involved ALNs [9, 13, 81, 84], and the negative predictive value (NPV) of ALN metastasis was 92.3% for tumor-positive internal mammary chain sentinel nodes [59]. Therefore, based on the current evidence, an elevated risk of IMLN metastasis appears to be associated with a medial or central location, younger age (< 50 years old), triple-negative hormone receptor status or positive HER-2 expression, and involvement of ≥ 4 ALNs metastases, and high nuclear grade.

A recent study also indicated that nipple inversion and mammographic calcification were strongly associated with a higher rate of IMLN metastasis [85]. Other results have suggested that IMLN metastasis/recurrence was independently influenced by tumor size, especially for invasive tumors with a size of > 2 cm [50, 80, 85]. However, in a large series of 2269 Chinese breast cancer patients who underwent extended radical mastectomy, IMLN metastasis was independently associated with number of ALNs and age, but not tumor size [81]. These conflicting findings may be related to racial or regional differences. During the follow-up evaluations, 66.2% of the patients had other concurrent metastasis sites with the IMLN recurrence, and these patients had a median survival time of only 42 months, relative to the 63 months for patients with isolated IMLN recurrence. Other independent factors that might delay IMLN recurrence were a small tumor size and positive ER/PR expression [68].

Based on clinicopathological risk factors, Sun Yat-sen University Cancer Center found that IMLN metastasis was significantly correlated with tumor location, lymphovascular invasion (LVI) and pathological ALN (pALN) stage in multivariable analysis [86]. Based on the multivariable logistic regression, they developed a user-friendly and effective nomogram to a prediction model constructed for IMLN status in breast cancer, but compared to IM-SLNB, the false-negative (FN) rate of nomogram for the detection of IMLN disease was still higher than that of IM-SLNB (13.9% vs. 3.3%) [86, 87]. On further expansion of the sample size (444 patients in the training cohort and 180 patients in the validation cohort) and independent external validation cohorts from other hospitals, non-invasive nomogram still had a 34 and 7% FN rate in the training and validation cohorts, respectively [88]. Non-invasive prediction tool must be based on prospective, large-scale and multicenter clinical trials, that to select patients with high risk of IMLN metastasis to undergo tailored treatment strategies, while for low risk patients can omit IMLN surgery or irradiation.

## Conclusions

The results of various population-based studies (22,922/10925, MA.20, and DBCG-IMN) have supported the use of IMLN RT after surgery, based on the therapeutic effect and long-term cardiotoxicity of IMLN irradiation [12, 89, 90]. Furthermore, the National Comprehensive Cancer Network Guidelines (version 1.2016) updated the level of evidence and strength of the recommendation for IMLN RT [20]. However, 56% of cardiac events occur after 10 years [91], and the Early Breast Cancer Trialists’ Collaborative Group proved that non-cancer deaths increased for breast cancer patients with N1 disease who underwent regional LN irradiation [92]. Therefore, clinicians must still comprehensively and conservatively estimate the potential benefits of IMLN RT and the risks of any toxic reactions. This assessment should include tumor position, volume, and histopathological grading, heart and lung functions, any use of anthracycline/trastuzumab, and reasonable life expectations [2, 9, 40, 50, 80–85, 93, 94]. Patients with increased long-term radiation-induced cardiovascular risks in coronary heart disease patients is greater than that in the general population [95]. Breast cancer chemotherapy with anthracycline and targeted therapy with trastuzumab increases the risk of cardiovascular risk. Drug-induced myocardial injury along with radiation-induced endothelial injury and inflammatory cell infiltration may further contribute to the increased risk of ischemic heart disease. Therefore, superior diagnostic and/ or predicted tools should be developed. Further studies are needed to examine the genomic and radiomic characteristics of breast cancer patients who have an elevated risk of IMLN metastasis, which will allow physicians to develop personalized and reasonable IMLN surgery or irradiation strategies to avoid over-/under-treatment.

## Data Availability

The datasets/or images are available from the corresponding author on reasonable request.
